# Editorial: Consanguinity and rare genetic neurological diseases

**DOI:** 10.3389/fneur.2025.1494253

**Published:** 2025-02-10

**Authors:** Suzanne Lesage, Mustafa A. Salih

**Affiliations:** ^1^Sorbonne Université, Institut du Cerveau - Paris Brain Institute - ICM, Institut National de la Recherche Médicale-U1127, Centre National de la Recherche Scientifique-UMR7225, APHP, Paris, France; ^2^King Abdulaziz City for Science and Technology (KACST), Riyadh, Saudi Arabia

**Keywords:** rare neurological diseases, mendelian inheritance, consanguinity, recessive inheritance, next-generation sequencing

Consanguinity and Rare Genetic Neurological Diseases Rare diseases are, by definition, those that affect a minor proportion of the population. A prevalence of <0.05% is considered as a rare disease by the European Union while in the USA, a condition affecting fewer than 200,000 people is considered rare. Rare autosomal recessive disorders are known to be exacerbated with consanguineous parents (1). Consanguineous marriages account for <20% to more than 50% of all marriages in Arab countries spanning from North Africa to the Middle East and Western Asia (2). Advancements in sequencing technologies, like whole exome or genome sequencing have accelerated gene discovery. These modern genetic tools can be useful to inform the genetics of consanguinity with the identification of novel disease alleles, hypomorphic alleles, and founder alleles (3–5). This Research Topic was designed to provide a platform for researchers to share their knowledge on rare genetic neurological disorders in consanguineous families with a special emphasis underlying underrepresented populations. Of the 11 manuscripts initially submitted to the journal by international researchers, eight were considered suitable for publication, after a thorough peer-review process. They included three original research articles, three case reports, one brief research report, and an erratum. Herein, we will present a short summary of each submitted article.

Xu et al. screen 34 patients with Wilson's disease (WD), a rare autosomal recessive disease characterized by hepatic, neurological and psychiatric symptoms. The hallmark of this disorder is an excessive accumulation of copper in the liver and the brain. WD is caused by mutations in the copper-transporter, *ATP7B*. The authors identify 25 potentially pathogenic variants in the *ATP7B* gene, five of which are novel. Renal biopsy performed in a patient with predominant renal involvement shows a thinning of the glomerular basement membrane, expanding the clinical spectrum of WD.

In a three-generation Chinese family with dominant hereditary spastic paraplegia (HSP), by using whole exome sequencing, Li et al. find a novel missense variant in an uncovered *ITPR1* gene shared by all the three affected family members and two young asymptomatic relatives. HSP is a group of rare neurodegenerative diseases of the corticospinal tract, predominantly characterized by lower limb weakness and spasticity. To date, more than 80 loci and 60 genes have been associated with HSPs. The novel HSP-associated gene, *ITPR1* is already known to be implicated in various conditions, such as SCA 15, 16, and 29, and the Gillespie syndrome.

This study expands the mutational spectrum of the *ITPR1* gene and its associated clinical heterogeneity, and the broad range of phenotypes associated with HSP. However, in absence of clear prediction of the variant pathogenicity and of functional validation *in vivo* and in engineered cells, these findings should be taken with caution.

Kleefstra syndrome (KLEFS) is a rare inherited neurodevelopmental disorder characterized by intellectual disability, neuropsychiatric anomalies, language and motor delays, and craniofacial abnormalities. It is a dominant condition caused either by microdeletions or a mutation in *EHMT1* (KLEFS1) or mutations in *KMT2C* (KLEFS2). To date, 13 patients with KLEFS2 have been reported. Yang et al. identify five additional unrelated Chinese patients with *KMT2C* mutations using exome sequencing. Herein, they present detailed clinical and phenotype information on these five patients carrying five undescribed heterozygous *KMT2C* mutations, expanding the mutational spectrum of KLEFS2. Summarizing the genotypes, phenotypes and clinical features of all 18 KLEFS2 cases, the authors conclude that patients with *KMT2C* mutations present with a wide range of phenotypic defects and a very large variable phenotype, with no significant genotype phenotype correlations.

Chen et al. describe a patient with a rare autosomal recessive primary microcephaly type 2 (MCPH2). To date, 30 mutated genes corresponding to MCPH1-MCPH30 have been reported, with MCPH2 associated with *WDR62* mutations, the second most frequent type of MCPH. A comprehensive clinical assessment including brain magnetic resonance imaging (MRI), electroencephalogram (EEG) and genetic analysis are conducted to evaluate the patient's condition. The patient born to consanguineous parents from China exhibits classic symptoms related to MCPH2, such as a microcephaly, intellectual disability, speech impairment, epilepsy, unilateral cerebral dysplasia and unrelated to the condition, limb deformities. Whole exome sequencing identifies a novel splice site mutation in the *WDR62* gene causing a significant reduction of the protein stability.

Mitochondrial encephalomyopathy, lactic acidosis and stroke-like episodes (MELAS) is one of the most common maternally inherited mitochondrial disease, caused by mutations in the mitochondrial genome. The most common MELAS mutation is the mitochondrial DNA (mtDNA), m.3243 A>G of tRNALeu (UUR) gene. In a case report, Zhao et al. describe a young woman with MELAS syndrome and autoimmune abnormalities. The patient presented with stroke-like episodes as first symptoms. She exhibits aggravated headache, blurred vision and diplopia, a history of diabetes and hearing loss, elevated blood levels of lactate and myogenic damage on muscle biopsy. The patient is positive for antinuclear antibodies and anti-mGluR5. However, the authors conclude that the association of MELAS syndrome with autoimmune disorders is not clear.

Zhu et al. report a Chinese young boy with Vulto-van Silfhout-de Vries (VSVS) syndrome followed over 2 years. The patient presents symptoms/signs as previously described for this condition: moderate global developmental delay and intellectual disability, severe language impairment, autism spectrum disorder, sleeping dysfunction, generalized seizures, imbalanced gait, increased pain threshold and undescribed recurrent respiratory infections. Whole exome sequencing identifies a *de novo* heterozygous pathogenic missense variant in the *DEAF1* gene, located in the SAND domain. The review in the literature worldwide identifies 35 individuals with 28 different *de novo* pathogenic variants in *DEAF1*- related VSVS. All the pathogenic variants identified are missense variants and located in the SAND functional domain of the DEAF1 protein. The majority of the 35 patients have the main characteristics related to VSVS, including juvenile age at onset (median age, 7.1 years, range 1.3–38 years), different levels of global developmental delay and intellectual disability, severe expressive language disorder, behavioral problems, autistic spectrum disorder, somnipathy, and increased pain threshold.

Romero et al. present a case involving two sisters with unexplained neurological symptoms and signs, such as refractory generalized seizures evolving into dysarthria, dysphagia, ataxia, cognitive decline, psychiatric symptoms in one of the two siblings, and abnormal electroencephalogram consistent with epilepsy. WES sequencing reveals the presence of a heterozygous nonsense variant in *AARS*, maternally transmitted and a heterozygous missense variant in *CACNA1A* gene, paternally transmitted. Both genes have previously been associated with diverse neurological conditions, including epileptic encephalopathy. The authors hypothesize that a dual mutation in the two genes, *AARS* and *CACNA1A* could explain the fast progression of symptoms and deterioration.

In conclusion, these studies enhance our understanding of the genetic heterogeneity associated with rare neurological disorders and highlight the pivotal role of genetic testing, diagnosis and managing of these conditions. This Research Topic would not have been possible without the contribution of all the authors listed, and their substantial and precious work. We also thank the staff of Frontiers in Neurology for their dedicated assistance and support.
